# One-Step Green Synthesis of Isoeugenol Methyl Ether from Eugenol by Dimethyl Carbonate and Phase-Transfer Catalysts

**DOI:** 10.3390/molecules29020551

**Published:** 2024-01-22

**Authors:** Zhihai Zhang, Yin Gong, Xinru Xue, Mengshuang Hu, Min Zhou, Yao Zhao, Zhiqiang Hu

**Affiliations:** College of Pharmacy and Chemistry & Chemical Engineering, Taizhou University, 93 Ji Chuan Road, Taizhou 225300, China; zzhwang520@outlook.com (Z.Z.); x16939351932023@163.com (X.X.); zmshizhoumin@163.com (M.Z.);

**Keywords:** green chemistry, DMC, phase transfer catalyst, polyethylene glycol 800, one-step synthesis

## Abstract

In this paper, the green synthesis of isoeugenol methyl ether (IEME) from eugenol by *O*-methylation and isomerization is completed using a one-step green process. In the methylation reaction, dimethyl carbonate (DMC) was used as a green chemistry reagent instead of the traditional harmful methylation reagents, in accordance with the current concept of green chemistry. The phase transfer catalyst (PTC) polyethylene glycol 800 (PEG-800) was introduced into the isomerization reaction to break the barrier of difficult contact between solid and liquid phases and drastically reduce the reaction conditions by shortening the reaction time and reducing the alkalinity of the reaction system. The catalytic systems for the one-step green synthesis of IEME were screened, and it was shown that the catalytic system “K_2_CO_3_ + PEG-800” was the most effective. The effects of reaction temperature, n(DMC):n(eugenol) ratio, n(PEG-800):n(eugenol) ratio, and n(K_2_CO_3_):n(eugenol) ratio on eugenol conversion, IEME yield, and IEME selectivity were investigated. The results showed that the best reaction was achieved at a reaction temperature of 140 °C, a reaction time of 3 h, a DMC drip rate of 0.09 mL/min, and n(eugenol):n(DMC):n(K_2_CO_3_):n(PEG-800) = 1:3:0.09:0.08. As a result of the conversion of 93.1% of eugenol to IEME, a yield of 86.1% IEME as well as 91.6% IEME selectivity were obtained.

## 1. Introduction

It is estimated that global demand for flavors and fragrances in 2013 amounted to USD 16 billion. Most of these flavors and flavor compounds are obtained through chemical techniques, with only a small portion of the demand being met through microbial sources [1]. The green synthesis of flavor and fragrance compounds has proved popular and is widely cited [2]. Phenolic ethers are widely used compounds that dominate the food and cosmetic industries due to their unique properties in flavors and fragrances [3]. Isoeugenol methyl ether (IEME), as a phenolic ether flavoring, is an important phenolic ether green monomer flavoring that is also used as a food additive and flavor enhancer [4]. Meanwhile, IEME has no adverse effects on the skin or internal organs in pathology studies and therefore can be widely used in daily toiletries [5]. Currently, IEME preparation processes are all biologically derived from various essential oils, but IEME is not currently available for commercial use due to its extraction process, which does not meet market demands [6,7].

The chemical preparation of IEME from eugenol involves two experimental steps, namely *O*-methylation and allylbenzene isomerization reactions.

### 1.1. O-Methylation

*O*-methylation of phenolic compounds is an important synthetic method in organic chemistry and has high applicability in the synthesis of fragrances [8]. The hydroxyl group of phenol reacts with a methylation reagent and undergoes an *O*-methylation reaction to form aryl methyl ethers [9]. Methylation reactions are carried out using a wide range of methylation reagents, such as dimethyl sulphate, methyl halide, methanol, etc. [10]. High toxicity, environmental pollution, and low reaction efficiency are the disadvantages of the methylation reagents listed above [11,12]. In recent years, dimethyl carbonate (DMC) has been reported as an environmentally sustainable compound and a new type of green chemical material [13]. It can be used as a non-toxic solvent, an effective fuel additive, and a synthetic intermediate in a variety of medical, pharmaceutical, and chemical applications [14]. Furthermore, DMC is an environmentally friendly alternative to highly toxic and hazardous compounds and is often used as a substitute for dimethyl sulfate and halogenated hydrocarbons in phenol-ether *O*-methylation reactions [15,16]. *O*-methylation of phenol using DMC has been reported using alkali base, tertiary amina, phosphonium salts, basic zeolites, alumina, and alumina–silica as base catalysts [17,18].

### 1.2. Allylbenzene Isomerization

**Figure 1:**
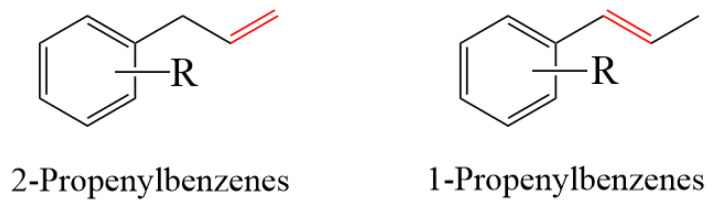
Allylbenzene isomerization.

The isomerization reaction of 2-propenylbenzene to 1-propenylbenzene (as presented in Figure 1) has been used in a wide range of applications in flavors and fragrances, cosmetics, pharmaceuticals, and materials chemistry, as well as a synthetic intermediate to manufacture complex products [19,20]. For most of the isomerization reactions, strong bases such as potassium hydroxide (KOH) or sodium hydroxide (NaOH) are usually used as catalysts [21]. However, when a base is used as a catalyst for an isomerization reaction, the conditions are usually more severe, requiring high temperatures and a longer reaction time. Despite the fact that the allylbenzene isomerization reaction condition is demanding, it is still of great academic and industrial importance, providing an efficient and economical route to the synthesis of chemical compounds [22].

Multiphase reactions are always difficult to conduct, owing to the immiscibility of the phases. However, with the help of an amphiphilic agent, which is soluble in both aqueous and organic phases, such reactions are now possible [23]. The phase transfer catalyst (PTC) allows transferring substances from one system to another in a chemical reaction between the two non-miscible, heterogeneous systems, which itself has both functional sites to get solubilized in both systems [24]. Typical PTCs are quaternary ammonium salts, crown ethers, quaternary phosphonium salts, etc. [25,26]. Exploring the discovery that polyethylene glycol (PEG) is inexpensive, has minimal impact on humans and the environment, and can replace crown ethers as PTCs [27]. PEG has excellent biocompatibility, exhibits high activity in liquid–solid phase catalysis, and has good complexation with metal base ions [28,29]. PEG provides better water solubility and simpler post-processing than several other PTCs [30]. It has been reported that phenolic compounds can be *O*-methylated with DMC with various PTCs, such as crown ether (18-Crown-6), PEG, tetrabutylammonium bromide, etc. [31].

In this experiment, a green and efficient one-step synthesis of IEME was investigated. DMC, as a green chemical reagent, was used to replace the toxic and harmful traditional *O*-methylation reagent to make the synthesis route clean and environmentally friendly. Subsequently, the catalytic system for the one-step green synthesis of IEME was screened by comparing different combinations of catalyst and PTC to test their efficiency. Finally, the conditions of the reaction were optimized by testing various factors, including reaction temperature, catalyst dosage, PTC dosage, and DMC drip rate.

## 2. Results and Discussion

### 2.1. Catalytic System Categories

A one-step green synthesis of IEME with different combinations of catalysts and PTCs was examined in terms of eugenol conversion, yield, and selectivity of IEME. The one-step synthesis of IEME reaction conditions was as follows: the reaction temperature was 160 °C, the reaction time was 3 h, the DMC drip rate was 0.09 mL/min, and the ratio of reactants was n(eugenol):n(DMC):n(catalyst):n(PTC) = 1:4:0.1:0.1. The experimental results are shown in Table 1.

DMC as a methylation reagent can be directly selected from metal hydroxides, metal carbonates, etc., as catalysts [32]. The inorganic substances, such as KOH, KF, K_2_CO_3_, CH_3_COOK, Na_2_CO_3_, and NaOH, were used as catalysts for the one-step green synthesis of IEME. As shown in Table 1, the properties of these catalysts greatly influenced the experimental results. When KF, K_2_CO_3_, CH_3_COOK, and Na_2_CO_3_ were used as catalysts, the magnitude of basicity as judged by pKa was found to be greater for K_2_CO_3_, which had a better effect on the conversion of eugenol, reaching 89.7%. However, the effect of these catalysts on IEME yield and selectivity was minimal. It is worth mentioning that the selectivity of IEME seems to be the highest when strong bases, KOH and NaOH, are used as catalysts, reaching 83.6% and 70.8%, respectively. The main reason for this is because a more basic catalyst has a greater potential for facilitating isomerization reactions. However, the conversion of eugenol is low when using this kind of catalyst due to the fact that in the presence of strong bases, eugenol forms phenolic salts, which impede the methylation reaction [33]. In conclusion, the catalyst is more favorable for the conversion of eugenol when it is weakly basic, which means that it provides a favorable environment for *O*-methylation. A catalyst that exhibits a strong base nature favors IEME yield and selectivity, which means that this type of catalyst facilitates the isomerization reaction [34].

As can be seen from Table 1, the addition of different types of PTCs resulted in a significant increase in the yield and selectivity of IEME. A noteworthy detail is that when comparing different catalytic systems such as “KOH + 18-Crown-6”, we found that the “KOH + 18-Crown-6” catalytic system did not have a significant effect on the conversion of eugenol, which was due to the fact that KOH, as a strong alkali catalyst, had less effect on *O*-methylation. This phenomenon suggests that the addition of PTC has no effect on the *O*-methylation reaction, but for the “K_2_CO_3_ + 18-Crown-6” catalytic system, the effect of PTC is quite obvious, resulting in a substantial increase in the yield and selectivity of IEME. Subsequently, different classes of PTCs were examined, such as crown ether, TBAB, and PEG series. By comparison, we have learned that the addition of PTCs improves the yield and selectivity of IEME and facilitates the isomerization reaction. Further, PEG series PTCs provided the best catalytic results in terms of conversion of eugenol, yield and selectivity of IEME, convenience of post-experimental treatment, and price. During a comparison of PEG-400, PEG-600, and PEG-800 under the same experimental conditions, it was found that PEG-800 (eugenol conversion: 92.6%, IEME yield: 86.1%, and IEME selection: 93.0%) was superior to PEG-400 and PEG-600 in terms of eugenol conversion, IEME yield, and selectivity under the same experimental conditions. It is because PEG-800 has a larger molecular weight and longer molecular chains, which can be folded into helical and free-sliding chains, making it better able to complex with K^+^. This phenomenon is consistent with the experimental phenomenon that K_2_CO_3_ is completely soluble in PEG-800 at 130 °C, whereas higher temperatures are required for PEG-400 and PEG-600. Therefore, “K_2_CO_3_ + PEG-800” was chosen as the catalytic system for the one-step green synthesis of IEME from eugenol.

### 2.2. Single-Factor Influence

In this experiment, K_2_CO_3_ was used as the basic catalyst and PEG-800 was used as the PTC to investigate the effects of reaction temperature, the proportion of n(eugenol):n(K_2_CO_3_):n(eugenol):n(PEG-800) and DMC drip rate on the eugenol conversion, yield of IEME, and selectivity of IEME.

#### 2.2.1. Effect of Reaction Temperature on Reaction

**Figure 2:**
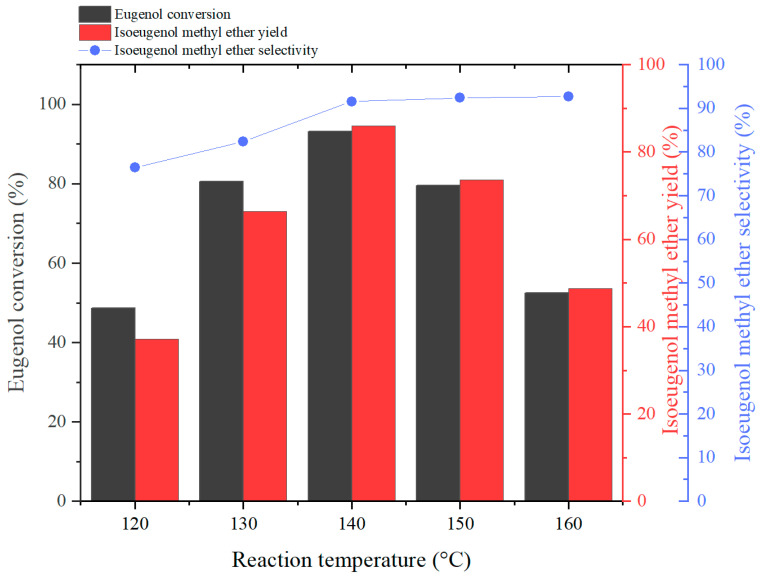
The effect of reaction temperature on Eugenol conversion, IEME yield and selectivity.

The effect of different reaction temperatures on the product yield was investigated by stipulating n(eugenol):n(DMC):n(K_2_CO_3_):n(PEG-800) = 1:4:0.09:0.08, and the experimental results were as follows:

As can be seen from Figure 2, the eugenol conversion and IEME yield were significantly affected by the reaction temperature. Firstly, as the reaction temperature increased from 120 °C to 140 °C, the eugenol conversion increased from 48.6% to 93.1%. Meanwhile, the IEME yield increased significantly (from 37.2% to 86.0%) with the reaction temperature. This is attributed to the activity of DMC, which is most active at temperatures between 130 and 140 °C [35]. As the reaction temperatures continue to rise between 140 °C and 160 °C, this results in a significant decrease in eugenol conversion (52.5%) and IEME yield (48.7%). This is due to the fact that DMC has a boiling point of 90 °C. If the reaction temperature is higher than 140 °C, the DMC will volatilize and not be able to react with the eugenol in the system, resulting in lower eugenol conversion and IEME yield [15]. However, the IEME selectivity showed an opposite trend with reaction temperature. With increasing temperature, the IEME selectivity continued to increase and remained in equilibrium after a reaction temperature above 140 °C. This phenomenon supports previous studies that found that high temperatures favor isomerization of allyl groups [21].

#### 2.2.2. Effect of n(Eugenol):n(K_2_CO_3_) Ratio on the Reaction

**Figure 3:**
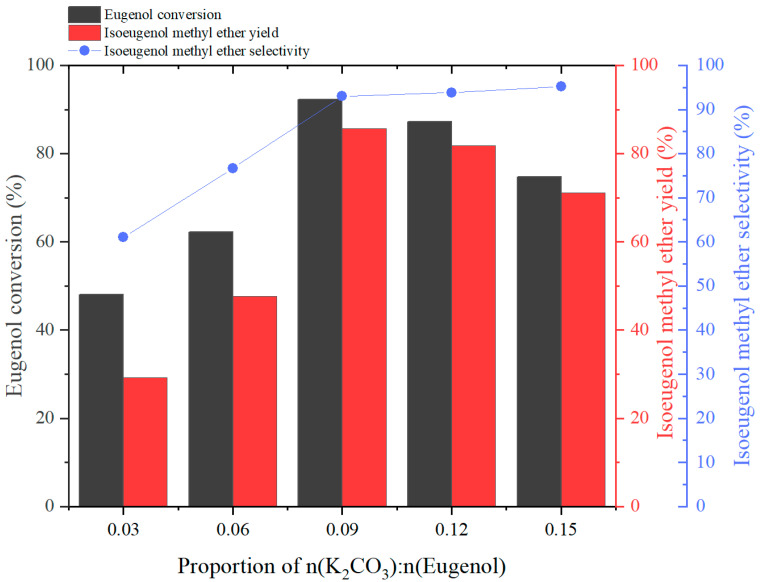
The effect of the proportion of n(K_2_CO_3_):n(Eugenol) on Eugenol conversion, IEME yield, and selectivity.

The effect of different amounts of K_2_CO_3_ on the yield of the products was investigated by stipulating n(eugenol):n(DMC):n(PEG-800) = 1:4:0.09, and the experimental results were as follows:

Figure 3 shows the effect of catalyst K_2_CO_3_ dosage on eugenol conversion, IEME yield, and selectivity. When the proportion of n(K_2_CO_3_):n(Eugenol) increased from 0.03 to 0.09, the eugenol conversion, IEME yield, and selectivity increased dramatically with the increase in proportion. The eugenol conversion yield reached 92.2%, while the IEME yield and selectivity were as high as 85.7 and 93.0%, respectively. However, it is worth mentioning that the eugenol conversion and IEME yield showed a decreasing trend as the proportion of n(K_2_CO_3_):n(eugenol) increased from 0.09 to 0.15, resulting in the eugenol conversion and IEME yield decreasing to 74.7% and 71.1%, respectively. This is due to the fact that weak base conditions favors *O*-methylation reactions in the presence of DMC [36]. However, the anomaly was observed, i.e., the IEME selectivity continued to increase as the proportion of n(K_2_CO_3_):n(eugenol) was increased, despite the eugenol conversion and IEME yield decreasing. This is due to the increase in this proportion, which leads to an increase in the basicity of the reaction system. The isomerization reaction is favored by a strong base [21].

#### 2.2.3. Effect of n(Eugenol):n(PEG-800) Ratio on the Reaction

**Figure 4:**
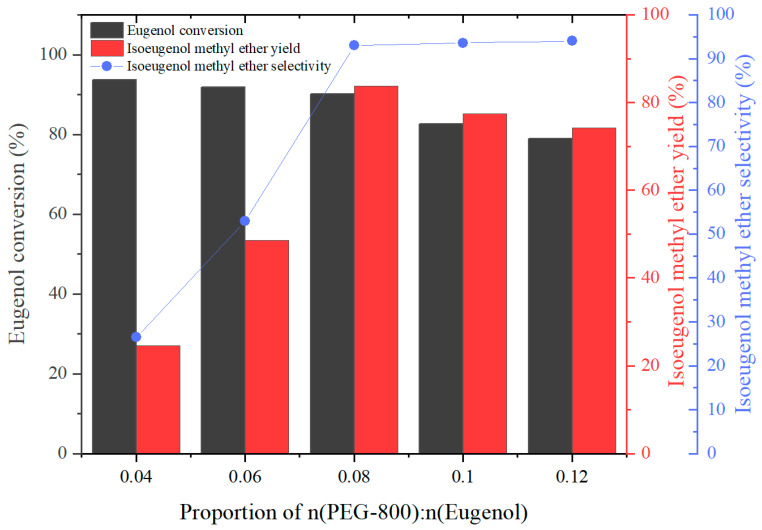
The effect of the proportion of n(PEG-800):n(eugenol) on eugenol conversion, IEME yield, and selectivity.

The reaction temperature for the one-step green synthesis of IEME was set at 140 °C. The effect of different dosages of PEG-800 on the product yield was investigated by stipulating n(eugenol):n(DMC):n(K_2_CO_3_) = 1:4:0.09. The experimental results were as follows:

As shown in Figure 4, the eugenol conversion decreased by increasing the proportion of n(PEG-800):n(eugenol). This was caused by the addition of PEG-800, which increased the viscosity of the reaction system, resulting in reduced contact between DMC and reducing eugenol conversion. The proportion of n(PEG-800):n(eugenol) increased from 0.04 to 0.12, the eugenol conversion decreased from 93.7% to 78.9%, and the IEME yield increased from 24.6% to 74.2% with the proportion increasing. However, the IEME selectivity continued to increase with the addition of PEG-800, i.e., the IEME selectivity increased to 94% when the proportion of n(PEG-800):n(eugenol) was increased to 0.12. This is due to the fact that in the presence of the PEG-800, the inorganic catalyst K_2_CO_3_ dissolves from the solid phase into the PEG-800 to form a liquid phase, which increases the chance of contact with eugenol methyl ether, which is also in the liquid phase. Thus, increasing the chance of the isomerization reaction occurring. It resulted in an increase in the IEME yield followed by a decrease. As the proportion of n(PEG-800):n(eugenol) increased from 0.04 to 0.08, the IEME yield showed an increasing trend, reaching a maximum of 83.8%. Subsequently, as the proportion of n(PEG-800):n(eugenol) continued to increase, the IEME yield reduced to 71.2% when the proportion reached up to 0.12.

A phenomenon that also deserves to be noticed is that the IEME yield and selectivity are not significant when the conversion of eugenol is high, with the proportion of n(PEG-800):n(eugenol) equal to 0.04, which demonstrates that the isomerization reaction is not significant under these conditions. However, as the proportion of n(PEG-800):n(eugenol) increased from 0.04 to 0.08, the IEME yield and selectivity increased. We deduce that the presence of PEG-800 greatly affected the isomerization reaction.

#### 2.2.4. Effect of n(Eugenol):n(DMC) Ratio on the Reaction

**Figure 5:**
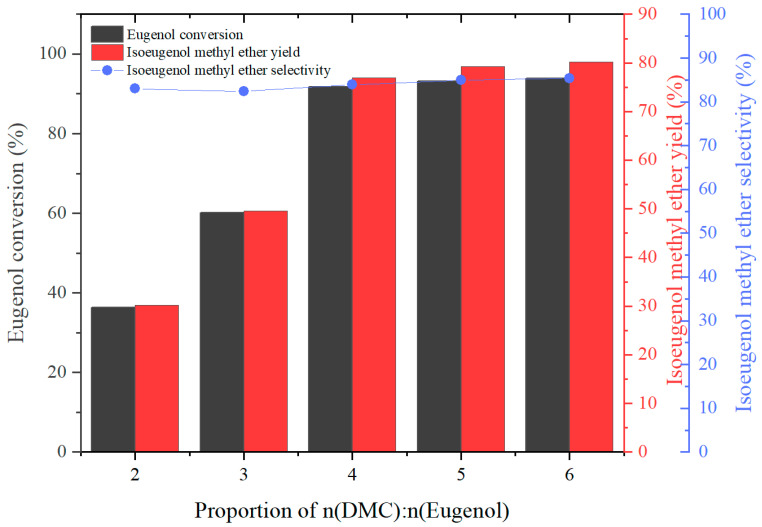
The effect of the proportion of n(DMC):n(eugenol) on eugenol conversion, IEME yield, and selectivity.

The effect of different DMC dosages on the product yield was investigated by stipulating n(eugenol):n(K_2_CO_3_):n(PEG-800) = 1:0.09:0.08, and the experimental results were as follows:

As we know from Figure 5, the proportion of n(DMC):n(Eugenol) directly affected the eugenol conversion. The eugenol conversion increased from 36.4% to 91.8% as the proportion of n(DMC):n(Eugenol) increased from 2 to 4. As the proportion of n(DMC):n(Eugenol) continued to increase, the conversion of eugenol and the IEME yield fluctuated up and down around 93.0% and 80%, respectively. However, the IEME selectivity has been maintained at around 83–85% as the proportion of n(DMC):n(Eugenol) varies. In conclusion, the proportion of n(DMC):n(Eugenol) had no significant effect on the IEME yield and selectivity but a more significant effect on the eugenol conversion.

#### 2.2.5. Effect of DMC Drip Rates on the Reaction

**Figure 6:**
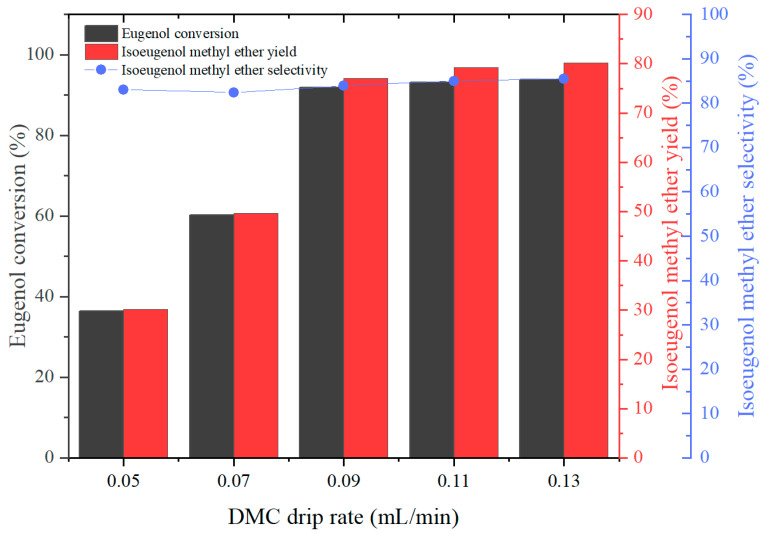
The effect of the proportion of DMC drip rate on eugenol conversion, IEME yield, and selectivity.

The effect of different drip rates of DMC on the IEME yield and selectivity was investigated by stipulating n(eugenol):n(K_2_CO_3_):n(PEG-800) = 1:0.09:0.08, and the experimental results were as follows:

As we investigated the effects of various factors on the conversion of eugenol, the IEME yield, and selectivity throughout the experiment, we learned that the drip rates of DMC had a significant impact on the reaction when we examined the effects of different factors. As we can see from Figure 6, when the drip rates of DMC increased from 0.05 to 0.13 mL/min, the conversion of eugenol and the yield and selectivity of IEME were significantly enhanced. An understanding of the reasons for this trend can be gained from the experimental phenomena. When the drip rate of DMC was excessively fast, it would make the reaction temperature lower and not reach the temperature required for the one-step synthesis reaction condition. At the same time, the excessively fast dripping rate of DMC would lead to the by-product methanol not being removed from the reaction system in time, which would inhibit the *O*-methylation reaction, thus affecting the continuous occurrence of the subsequent reaction. A consistently lowered DMC drip rate did not have any significant effect on the conversion of eugenol, yield, or selectivity of IEME but rather increased the cost of IEME production. So that the DMC drip rate is optimal at 0.09 mL/min.

## 3. Experimental Section

### 3.1. Materials

Eugenol (99%), Dimethyl Carbonate (DMC), Polyethylene Glycol 600 (PEG-600), and PEG-800 were purchased from Sahn Chemical Technology (Shanghai) Co. Ltd. (Shanghai, China) Polyethylene Glycol 400 (PEG-400) was purchased from McLean Biochemical Technology Co. (Shanghai, China). Additionally, Potassium Carbonate (K_2_CO_3_), Potassium Hydroxide (KOH), Potassium Fluoride (KF), Potassium Acetate (CH_3_COOK), Sodium Carbonate (Na_2_CO_3_), Sodium Hydroxide (NaOH), and Ethyl Acetate (EtOAc) were purchased from Sinopharm.

### 3.2. Methods

#### 3.2.1. Synthesis Process

**Figure 7:**
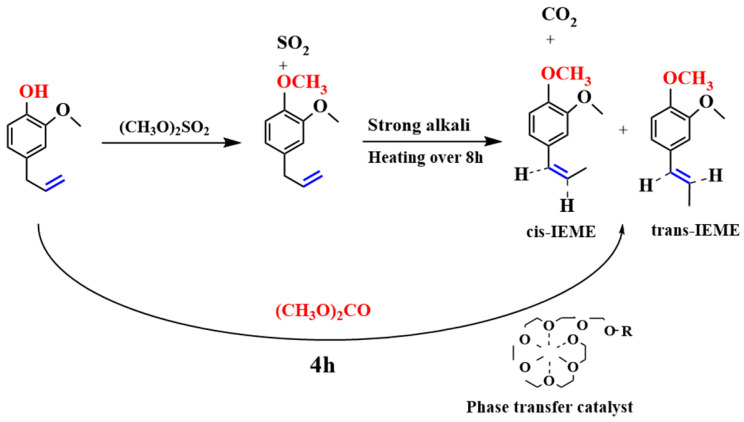
One-step synthesis of IEME.

**Figure 8:**
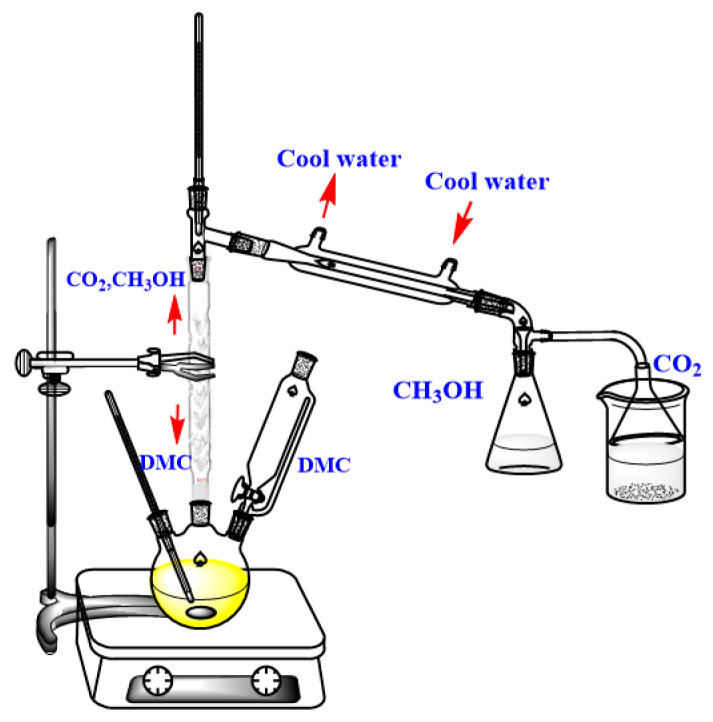
Process flow diagram for the synthesis of IEME.

The one-step process for the synthesis of IEME via the green chemical reagents DMC and PTC (PEG-800) is shown in Figure 7. A 250 mL three-necked flask equipped with a distillation column, thermometer, dropping funnel, and magnetic stirrer was used to conduct the experiment (as presented in Figure 8). Appropriate amounts of alkali catalyst K_2_CO_3_ and PTC PEG-800 were first added to a three-neck flask and preheated until K_2_CO_3_ was completely dissolved in PEG-800. The reaction reagent, eugenol, was then added to a three-necked flask. An external thermometer, distillation column, dropping funnel and condenser tubes were installed into the experimental setup while ensuring that it was gas-tight. Before the drop-wise addition of the DMC reagent, N_2_ was vented at atmospheric pressure to prevent the oxidation of eugenol. After the eugenol was heated to a preset temperature and stirring was carried out, DMC was added slowly and dropwise to allow full contact between the DMC and eugenol during the *O*-methylation process. It is important to note that there are two concerns regarding the experimental phenomenon. During the reaction, CO_2_ will be produced as a by-product, which will bubble up and be absorbed in the calcium hydroxide solution. Another phenomenon is that methanol is produced as a by-product, which inhibits eugenol *O*-methylation. Detaching methanol from the reaction system favors the reaction and improves the utilization of DMC. Considering the boiling points of DMC (90–91 °C) and methanol (64.8 °C), the utilization of DMC was improved by using a distillation column to separate methanol from DMC, which condensed and continued to react with eugenol. Finally, the *O*-methylation reaction is complete when bubbles are no longer observed, and the reaction is maintained at 140 °C for 0.5 h. After the reaction, the acidity of the solution was adjusted by 0.1 mol/L dilute hydrochloric acid to make the pH less than 7. The solution was extracted three times with ethyl acetate, and the organic reagents were dissolved in ethyl acetate. The organic phase was subsequently washed three times with distilled water. A dispensing funnel is used to separate and obtain the organic phases of a solution. The organic solvent was evaporated under reduced pressure to obtain the product, and the sample was analyzed to calculate the eugenol conversion, IEME yield, and selectivity. The sample was analyzed by gas chromatography (GC).

#### 3.2.2. GC Analysis

A GC-9720 GC equipped with a flame ionization detector (FID) and an HP-INNOWAX capillary (non-polar) was employed to analyze the samples. The GC column box, injector, and detector were all heated to 270 °C, 265 °C, and 270 °C, respectively, using nitrogen as the carrier gas at a split ratio of 40:1, a flow rate of 0.85 mL/min, and a volume of injection of 0.6 μL. GC begins at 120 °C, is held at that temperature for 1 min, increases to 265 °C at 10 °C/min, and is held at 265 °C for 2 min. The yield of IEME in each experiment was determined from the amount of IEME in the components analyzed by GC using standard curve analysis.

#### 3.2.3. IEME Yield Analysis

The formula for the eugenol conversion, yield, and selectivity of IEME is given below:

(1) $$Eugenol\;conversion\;(\%)=\frac{n(eugenol\;(initial))-n(eugenol\;(final))}{n(eugenol)}\times 100\%\;$$

(2) $$IEME\;yield\;\left(\%\right)=\frac{m\times w}{M\times n}\times 100\%$$

(3) $$IEME\;selectivity\;(\%)=\frac{\left[Isoeugenol\;methyl\;ether\;yield(\%)\right]}{\left[Eugenol\;conversion(\%)\right]}\times 100\%$$

where *m*: mass of isoeugenol methyl ether, g;

*w*: content of IEME in the product, %, detected by GC;
*M*: relative molecular mass of IEME, g/mol;
*n*: theoretical molar value of IEME, mol.

#### 3.2.4. Reaction Mechanism

**Figure 9:**
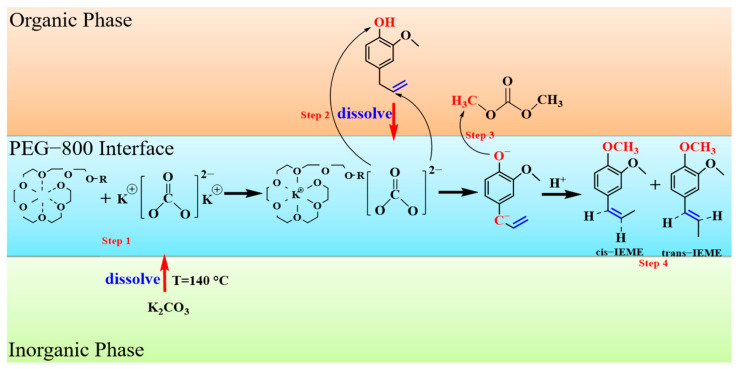
Reaction mechanism diagram.

The reaction mechanism for the one-step green synthesis of IEME from eugenol is assumed to be divided into four stages (as presented in Figure 9). Step 1: K_2_CO_3_ is first added to the PEG-800 solution and heated to 140 °C, at which point the K_2_CO_3_ will be completely dissolved in the PEG-800 solution. The polymer PEG can be folded into a helical and free-sliding chain, which allows PEG to complex with the K^+^ in potassium carbonate; thereby, the solid phase K_2_CO_3_ is able to dissolve in the organic phase PEG [37,38]. As the K^+^ ions in K_2_CO_3_ are enclosed by PEG, the CO_3_^2−^ ions are exposed during the dissolution of K_2_CO_3_ in PEG. Step 2: Eugenol, as an organic reagent, is soluble in the organic phase polymer PEG. The phenolic hydroxyl group in eugenol reacts with CO_3_^2−^ to form ArO^−^ and CO_2_ as by-products [39]. Meanwhile, CO_3_^2−^, in the presence of PEG, can react with α-H in an allyl group (usually a strong base is required) to form C^−^ ion. Step 3: DMC reagent is added to the reaction for electrophilic methylation. DMC generates a C^+^ ion at elevated temperatures and reacts with the active O^−^ on the eugenol to produce the corresponding phenol ether. When the inorganic salt K_2_CO_3_ dissolves, the exposed CO_3_^2−^ ion reacts with α-H on the ally group to form a C^−^ ion. This creates an empty P-orbital on the α-C, which forms a P-Π conjugation with the Π-bond on the benzene ring, which is less stable than the Π-Π conjugation [40]. Thus, under heated conditions, allyl group isomerization forms a 1-propenyl group, which is thermodynamically more stable than the allyl group forming a P-Π conjugation with the benzene ring [41]. Step 4: After isomerization, the C^−^ ion on the 1-propenyl group reacts with the H^+^ ion to eventually produce IEME.

## 4. Conclusions

In this study, the one-step green synthesis of IEME from eugenol via *O*-methylation and isomerization was investigated. Firstly, a condensation reflux process was used to improve DMC utilization and significantly increase product yields by separating DMC and by-products in a distillation column. Secondly, through the screening of catalyst systems, the “K_2_CO_3_ + PEG-800” catalytic system was found to be the most efficient catalyst for the one-step synthesis of IEME. Based on the results of the experiments conducted, it was found that weak bases favored *O*-methylation reactions, strong bases favored isomerization reactions, and the application of PTCs allowed isomerization to occur even in weakly basic conditions. Furthermore, PTCs have shortened the isomerization reaction time and enabled the reaction conditions to be carried out at low temperatures instead of the previous high temperatures. Subsequently, the reaction factors such as reaction temperature, n(eugenol):n(K_2_CO_3_) ratio, n(eugenol):n(PEG-800) ratio, n(eugenol):n(DMC) ratio, and DMC droplet rate were optimized, and it was found that at the reaction temperature of 140 °C and the DMC drip rate of 0.09 mL/min, n(eugenol):n(DMC):n(K_2_CO_3_):n(PTC) = 1:3:0.09:0.08, the eugenol conversion, IEME yield, and selectivity were optimized and the results were 93.1%, 86.1%, and 91.6%, respectively.

## Figures and Tables

**Table 1:** Comparison of IEME yield and selectivity over different catalytic system.

Catalytic System	Catalyst	Eugenol Conversion (%)	IEME Yield (%)	IEME Selectivity (%)
Inorganic catalyst	KOH	42.1	35.2	83.6
	KF	11.5	7.4	64.3
	K_2_CO_3_	89.7	10.9	12.2
	CH_3_COOK	7.4	0.6	8.1
	Na_2_CO_3_	65.2	3.3	5.0
	NaOH	34.7	24.6	70.8
Inorganic catalyst and PTC	K_2_CO_3_ + 18-Crown-6	88.2	78.6	89.1
	KOH + 18-Crown-6	45.6	40.1	87.9
	K_2_CO_3_ + TBAB	80.7	65.6	81.3
	K_2_CO_3_ + PEG-400	84.2	71.3	84.7
	K_2_CO_3_ + PEG-600	88.9	77.6	87.3
	K_2_CO_3_ + PEG-800	92.6	86.1	93.0

Reaction conditions: reaction temperature 160 °C, reaction time 3 h, DMC drip rate 0.09 mL/min, ratio of reactants: n(eugenol):n(DMC):n(catalyst):n(PTC) = 1:4:0.1:0.1.

## Data Availability

Data are contained within the article.
